# EEG Microstates are Not Associated with Psychotic Symptoms, Neurocognitive Domains or Psychosocial Functioning in Adolescents and Young Adults with Clinical High Risk for Psychosis

**DOI:** 10.1007/s10548-026-01255-x

**Published:** 2026-10-10

**Authors:** Matthias Liebrand, Carla Wedekind, Angelos Katsarakis, Sarah Diezig, Chantal Michel, Frauke Schultze-Lutter, Michael Kaess, Daniela Hubl, Thomas Koenig, Jochen Kindler

**Affiliations:** 1 University Hospital of Child and Adolescent Psychiatry and Psychotherapy, University of Bern, Bern, Switzerland; 2 Translational Research Center, University Hospital of Psychiatry and Psychotherapy, University of Bern, Bern, Switzerland; 3 Department of Psychiatry and Psychotherapy, Medical Faculty, University Hospital Düsseldorf, Heinrich-Heine-University, Düsseldorf, Germany; 4 Department of Psychology, Faculty of Psychology, Airlangga University, Surabaya, Indonesia; 5 Hospital of Child and Adolescent Psychiatry and Psychotherapy Baselland, Liestal, Switzerland

**Keywords:** EEG, Microstates, Clinical high-risk, Psychosis, Correlation, Clinical symptoms

## Abstract

EEG microstates are brief and discrete topographic patterns of electrical brain activity. Microstates last for 60–120 milliseconds, are thought to represent transient, quasi-stable neural networks, and traditionally four distinct classes (A-D) are distinguished. One of the core concepts of present neuroscience is that neuroimaging is able to capture meaningful networks of brain activity, which can be translated into specific cognitive functions. Following this idea, each microstate might correspond to specific core brain functions. Importantly, these cognitive functions are impaired in mental disorders. Clinical high-risk (CHR) refers to a state associated with an increased risk of developing psychosis. Previous research has demonstrated consistent microstate alterations in CHR individuals (especially in class D), as well as in psychosis. However, the neurobiological and functional meaning of microstate alterations in CHR populations is still elusive. Since the CHR state is associated with reduced neurocognition (e.g., processing speed, verbal memory, executive functions) in combination with specific clinical symptoms and impairments in psychosocial functioning, relationships between resting-state microstates and these domains were examined in a large cross-sectional cohort of CHR individuals (*n* = 100). Contrary to our expectations, no significant correlations were found for these clinical measures. The finding suggests that while microstate parameters may differ between CHR individuals and controls, these alterations do not seem to directly reflect the neurobiology of clinical symptoms or neurocognitive and psychosocial functions. EEG microstate deviations as candidate trait biomarker therefore require further investigation and validation in longitudinal studies to establish their stability and predictive value.

**Supplementary Information:**

The online version contains supplementary material available at https://doi.org/10.1007/s10548-026-01255-x.

## Introduction

While many somatic disorders are fairly well understood in their pathophysiology, most psychiatric disorders, including psychosis and schizophrenia-spectrum disorders, still lack fundamental understanding (Nour et al. 2022). Further insight into mental disorders and their pathophysiological causes is crucial to pave the way for supporting patients in developing new and efficient treatments. This is even more true for schizophrenia, as prevalence worldwide nearly has doubled between 1990 and 2019 (14 to 24 million individuals), thereby being the third most prominent mental disorder leading to disability adjusted life years (DALYs) (GBD 2019 Mental Disorders Collaborators 2022). For psychosis, a clinical high-risk (CHR) state has been proposed to identify individuals at increased risk who present with attenuated psychotic symptoms (subthreshold symptoms without full conviction) and/or brief limited intermittent psychotic symptoms (transient episodes of fully psychotic symptoms), often accompanied by functional decline. Around 25% of CHR individuals are expected to transition to full psychosis within three years and 35% within ten years (Salazar de Pablo et al. 2021), while others remit, continue to experience subthreshold symptoms, or develop other psychiatric disorders. In the last two decades substantial research efforts have been invested in characterizing and understanding the CHR state, entailing the hope to prevent schizophrenia-spectrum disorders in intervening even before the first manifest psychotic episode emerges. Resting-state measures are a promising approach to advancing our comprehension and classification of the CHR state, as they are particularly well suited to characterizing network dynamics which are known to be disrupted in this high-risk stage (Xu et al. 2024; Paton et al. 2025). Electroencephalography (EEG) microstates are suggested to be a promising resting-state biomarker, as they have reliably been shown to be altered in CHR individuals (Andreou et al. 2014; de Bock et al. 2020; Luo et al. 2020; Liebrand et al. 2024, 2026) and in full-blown psychosis (da Cruz et al. 2020; Murphy et al. 2020; Vass et al. 2025). EEG microstates are short temporal periods (60–120 ms), in which the scalp global field remains quasi-stable, before rapidly transitioning to another topographic state (Michel and Koenig 2018). These quasi-stable states likely reflect simultaneous activity of large-scale neuronal networks and have been proposed to correspond to basic principles of information processing (Koukkou and Lehmann 1983). Overall, microstates explain a substantial amount (65–84%) of total EEG data variance (Michel and Koenig 2018). They show good reliability and consistency across analytic methods (Khanna et al. 2014; Liu et al. 2020; Popov et al. 2023; Kleinert et al. 2023). In microstate research the parameters “coverage” (percentage of time covered by a microstate class), “duration” (mean duration of a class in ms), “occurrence” (number of occurrences of a class per second) and “explained variance” (percentage of total EEG variance explained by microstate topographies) are commonly investigated.

A core assumption of EEG microstates research is that different topographies, labelled as classes, represent distinct functional states of brain activity, each potentially associated with specific cognitive processes (Michel and Koenig 2018). This is supported by findings that microstate classes can be linked to specific functional magnetic resonance imaging (fMRI) resting-state networks (Britz et al. 2010; Musso et al. 2010). Associations between distinct classes and their functional significance or underlying neural networks remain incompletely understood, but a recent review comprehensively summarized the existing knowledge (Tarailis et al. 2023). Although, up to seven classes have been distinguished, most existing studies focused on four canonical states, labelled A-D. Microstate A has been mostly linked to auditory and phonological processing (Korn et al. 2021) and associated with the auditory network (Britz et al. 2010; Custo et al. 2017). Microstate B may correspond to visual processing (D’Croz-Baron et al. 2021) and has been connected to the visual network (Britz et al. 2010; Custo et al. 2017). Microstate C might be associated with the default mode network (DMN), involved in “self-experience” (Custo et al. 2017; Bréchet et al. 2019), with the salience network (Britz et al. 2010), and might be relevant to the processing of personally significant information (Bréchet et al. 2019). Finally, microstate D may be involved in executive control including working memory (Kim et al. 2021) and attentional control (Ke et al. 2021), and might be located within the fronto-parietal network (Britz et al. 2010; Custo et al. 2017; Bréchet et al. 2019). It is important to note that these associations remain under debate, and further research is needed to elucidate the underlying structural and functional basis of microstates.

There are mixed findings about altered microstates in CHR individuals. On the one hand a reduction of coverage, (duration) and occurrence of class D (Luo et al. 2020; Liebrand et al. 2024) and increase of class C duration (Liebrand et al. 2024) has been observed in CHR vs. healthy control HC groups. Adding to this, the reduction of class D coverage and occurrence has been associated with conversion to psychosis (de Bock et al. 2020). On the other hand, a recent multi-site study investigating a large CHR sample (*n* = 460) found decreased temporal stability of microstates across classes to be associated with the CHR state and conversion to psychosis (Liebrand et al. 2026). Also, two other studies did not find effects in CHR individuals compared to HC (Andreou et al. 2014; Iftimovici et al. 2023). To date, only two studies investigated associations between microstates and disorder-related neural and/or functional aspects in CHR individuals. The study reporting reduced microstate temporal stability in CHR individuals, found a negative association between stability and positive symptoms, as measured with the positive symptom subscale of the Scale for the Assessment of Prodromal Symptoms (SOPS) (Liebrand et al. 2026). The other reported positive correlations between class A duration and the positive scale of the Positive and Negative Syndrome Scale (PANSS), between class A coverage/occurrence and the SOPS positive symptom subscale, and a negative correlation between the SOPS positive symptom subscale and class B duration (Andreou et al. 2014).

In manifest psychosis and schizophrenia-spectrum disorders, more is known about possible functional translation of microstates. Studies in patients with schizophrenia have shown that a decrease in microstate D duration was observed when patients experienced acute auditory verbal hallucinations (Kindler et al. 2011) and was associated with scores of paranoid-hallucinatory symptoms (Koenig et al. 1999). In a recent study investigating a large cohort of patients with first-episode psychosis (FEP), a negative correlation between class D duration and coverage and the Brief Psychiatric Rating Scale (BPRS), a rating scale of global psychopathology, was reported (Zhou et al. 2024). A meta-analysis of microstates in schizophrenia found the combination of increased class C occurrence and reduced class D duration to be a significant finding across studies of patients with schizophrenia and proposed that this pattern reflects a reduction of processes integrating contextual information (linked to class D) while saliency-oriented processes (associated with class C) are increased (Rieger et al. 2016). Summing up, evidence on the functional or neural meaning and foundation of aberrant microstates in CHR is very sparse and to our knowledge to date no study specifically focused on this topic.

Here, we aim to gain insight into associations between microstate parameters and general psychopathological and specific psychotic symptoms, neurocognitive correlates and psychosocial functioning, analyzing a large cohort of 100 CHR individuals. Previous studies found evidence for altered microstate parameters in classes C and D in CHR individuals (de Bock et al. 2020; Luo et al. 2020; Liebrand et al. 2024), which corresponds to findings in patients with schizophrenia-spectrum disorders (Rieger et al. 2016; da Cruz et al. 2020; Vass et al. 2025). Generally, a core assumption of cognitive neuroscience is that cognitive functioning depends on the coordinated interaction of distributed brain regions within functional networks (Park and Friston 2013). For example, attentional processes fundamentally depend on dynamic interactions within frontoparietal networks, including frontal regions and the parietal cortex. Following this idea, microstates as a potential measure of those networks should be linked to cognitive operations, especially so in psychiatric patients experiencing malfunction of cognitive processes. We therefore expected that disorder related symptoms, neurocognitive functioning and psychosocial functioning, which all can be expected to be related to cognitive functions, are specifically associated with the observed changes in classes C and D. Finally, given the limited evidence of microstate alterations in classes C and D in CHR to date, we also examined correlations between classes A and B and clinical symptoms, neurocognitive functions, and psychosocial functioning.

## Materials and Methods

### Sample

CHR individuals were help-seeking individuals who were referred to the Early Detection and Intervention Center for Mental Crises (FETZ (Michel et al. 2022) in Bern between 2011 and 2021. During this period, 514 individuals presented to the FETZ, 164 of them (31.9%) were classified as either CHR or FEP and received an EEG. CHR status was fulfilled (Fusar-Poli et al. 2013; Schultze-Lutter et al. 2015) when individuals met any CHR criteria according to the ultra-high-risk (UHR) and/or basic symptoms approach (see Supplementary Methods). Diagnoses were assessed by trained psychiatrists and psychologists at the FETZ (Michel et al. 2022). The CHR sample has been described previously (Liebrand et al. 2024). In contrast to the former sample, we did not include FEP patients nor clinical control (CC) subjects (individuals that presented to the FETZ, did not meet CHR criteria, and were diagnosed with any other psychiatric condition) as these groups were too small (FEP = 17, CC = 18 after exclusion of subjects with missing values) to perform valid correlational analyses. Furthermore, HC from the former sample were not included, as no data on clinical symptoms, cognitive testing or psychosocial functioning were available.

Exclusion criteria for CHR individuals were current substance abuse, clinically abnormal MRI and/or EEG, neurological disease with relevant impact on cognitive functions, IQ below 70 and abortion of the FETZ assessment. Subjects who either did not follow task instructions (eyes closed resting-state), showed more than 40% EEG artefacts or heavily distorted microstate topographies were also discarded from the analyses. Initially, 126 CHR individuals were recorded of which 26 CHR had to be excluded based on the above criteria, leaving a final sample of 100 CHR (Table 1). These displayed a low percentage of artifactual EEG which had to be removed (4.4%, standard deviation (SD) 4.3%). Because of missing values in the assessments of symptoms, cognitive functions and/or functioning in some CHR, the final analyzed sample included 93 CHR for analysis of correlations with symptom domains (65 unmedicated CHR), 93 CHR for cognitive domains (66 unmedicated CHR) and 98 CHR for global/social functioning (70 unmedicated CHR). The study was performed in agreement with the Declaration of Helsinki and all participants, and their parents in case of minors, gave informed consent. The study was approved by the ethical committee of the University of Bern (ID PB_2016-01,991).

### Psychopathological, Neuropsychological and Functional Assessments

Psychopathological correlates in terms of CHR-relevant symptoms were assessed using semi-structured clinical interviews. We used the Structured Interview for Psychosis-risk Syndromes (SIPS) (McGlashan et al. 2010), and the Schizophrenia Proneness Instrument, Child and Youth (SPI-CY) (Schultze-Lutter and Koch 2010; Fux et al. 2013) and Adult (SPI-A) (Schultze-Lutter et al. 2007) version, respectively. The SPI-A/SPI-CY rank basic symptoms (BS) on a severity scale according to the maximum frequency of their occurrence within the past 3 months ranging from 0 (absent = BS has not occurred in the past 3 months) to 6 (extreme = BS has occurred daily over sometime within the past 3 months). For the purpose of this analysis, ratings of 7 and 9 were recoded to 0 and 8 was recoded to 1 (see Supplementary Methods). The SIPS ranks positive and negative symptoms also from 0 (absent) to 6 (severe and psychotic) in its Scale of Prodromal Symptoms (SOPS). As domains for CHR symptoms we used the SOPS positive, negative, disorganization and general subscales, the COPER (cognitive-perceptive basic symptoms, 10 items from the SPI-A/SPI-CY reflecting broad BS) and COGDIS (cognitive disturbances, 9 items from the SPI-A/SPI-CY, also BS and reflecting subtle, subjective disturbances in thinking and perception) clusters and custom-selected clusters for perceptive and non-perceptive symptoms. The SOPS subscales, as well as COPER/COGDIS clusters, were used since these are established constructs in relation to the CHR syndrome. The clusters for perceptive and non-perceptive symptoms have been used before (Michel et al. 2022), and were implemented as we assumed perceptive symptoms to potentially be more psychosis-specific and non-perceptive symptoms rather unspecific to psychosis, which might be of relevance in terms of associations between microstate prameters and clinical symptoms. As correlate for the perceptive symptom cluster, a sum score of BS 13 and 14 from the SPI-A/SPI-CY and positive symptom P4 from the SOPS was used (see Supplementary Tables S1-S3 for items numbers and descriptions). For the non-perceptive symptom cluster the sum of BS 1–12, and SOPS items P1-P3 as well as P5 was used. This computation of perceptive and non-perceptive symptoms was adapted from Michel et al. (2022) and slightly modified (all raw scores for P1-P5 from the SOPS were used instead of only scores required for Attenuated Psychotic Symptoms (APS) or Brief Intermittent Psychotic Symptoms (BIPS), i.e., scores ≥ 3).

Neurocognitive functions were assessed with several scales including processing speed with the Digit Symbol Test (DST) from the Wechsler Intelligence Scale for Children – Fifth Edition (Petermann 2017); processing speed, visual attention, cognitive flexibility and set-shifting with the Trail Making Test (TMT) versions A & B (Partington and Leiter 1949); verbal fluency with the Regensburger Wortflüssigkeits-Test (RWT, Regensburg Verbal Fluency Test) (Aschenbrenner et al. 2000); crystallized verbal intelligence and semantic knowledge with the Peabody Picture Vocabulary Test (PPVT) (Dunn and Dunn 1997); verbal learning and memory with the Verbaler Learn- und Merkfähigkeitstest (VLMT, Verbal Learning and Memory Test) (Helmstaedter and Durwen 1990); and working memory with the Self-Ordered Pointing Task (SOPT) (Petrides and Milner 1982). The neurocognitive scales mentioned above were selected for use, as they were part of the standard FETZ protocol.

The level of psychosocial functioning was assessed using the Global Assessment of Functioning (GAF) scale (American Psychiatric Association 1987), the Social and Occupational Functioning Assessment Scale (SOFAS) (Goldman et al. 1992) and the Global Functioning: Social Scale (GF-S) (Cornblatt et al. 2007). The psychosocial functioning scales mentioned above were selected for use, as they were part of the standard FETZ protocol.

All data have been stored in RedCap software. Different measures were used for the scales and tests (GAF and SOFAS: raw values (1-100); GF-S: raw values (1–10); DST: raw values; VLMT (all subscales but Recognition) and PPVT: percentile ranks; VLMT Recognition: raw values; RWT: a mean of the percentile ranks over the fruits, sports, animals, s-words; SOPT: number of errors; TMT A and B: time in seconds).

### EEG Recording

EEG recording, preprocessing and microstate feature extraction were identical to Liebrand et al. (2024), with the exceptions that only the CHR group was analyzed, but not CC, FEP, and HC groups. Moreover, the ratio of classes C and D and transition probabilities (TP) were analyzed in addition. The ratio of classes C/D may be more informative than parameters of classes C and D alone, as both increases in class C and decreases in class D have been consistently observed in psychosis and schizophrenia (Rieger et al. 2016; da Cruz et al. 2020; Vass et al. 2025). TP from C to D and D to C were analyzed, as these have previously been shown to be altered in patients with schizophrenia (Rieger et al. 2016). The EEG was recorded with a 74-channel Nihon Kohden system (Neurofax 1100 amplifier, 500 Hz). Electrodes were placed according to an extension of the international 10–10 system (Ground: Fpz, reference: F3/F4). Impedance was kept below 20 kΩ. Electrocardiography (ECG) was additionally collected. During resting-state EEG recording (10 min) participants were comfortably seated in a separated room and instructed to close their eyes, stay awake, keep calm and relax. Every 120 s, they were told verbally to open their eyes for around 10–20 s and sit comfortably, in order to relax and stay awake. Participants’ vigilance or levels of drowsiness were not monitored during the resting-state recording.

### Preprocessing

For preprocessing of all data, we used Brain Vision Analyzer 2.2. Periods with eyes-open were excluded and noisy or otherwise bad channels were interpolated. Only for computation of an independent component analysis (ICA), data were filtered with a bandpass (2–20 Hz) and a notch (50 Hz) filter. ICA components for horizontal and vertical eyes movements, as well as ECG activity were visually identified and corrected. ICA-correction was then applied on unfiltered data. Subsequently, remaining artefacts were identified by visual inspection and removed. Finally, data were re-referenced to average reference.

### Microstate Feature Extraction

Microstates were analyzed using the Microstates Toolbox by Thomas Koenig (Version 1.2) implemented in EEGLAB (Delorme and Makeig 2004) in MATLAB R2020a. First, data were band-pass filtered (2–20 Hz) following previous microstate studies in schizophrenia (Koenig et al. 1999; Lehmann et al. 2005; Kikuchi et al. 2007; Kindler et al. 2011). Then global field power (GFP) was computed for each moment in time. All maps at momentary peaks of the GFP were selected and submitted to a modified k-means clustering algorithm (Pascual-Marqui et al. 1995). The k-means clustering was re-initialized 20 times, and the best solution was retained. The backfiring was limited to the moments of momentary GFP peaks, and the assignment of the remaining time was based on a nearest neighbor interpolation. Temporal smoothing was applied using the default settings of the Microstates Toolbox, with a minimum microstate duration of 20 ms. Microstate segments shorter than 20 ms were reassigned to the next best-fitting microstate based on topographic similarity. Polarity was disregarded during both clustering and backfitting, such that topographic maps with opposite polarities were considered equivalent. A set of four microstate classes was chosen, to be in line and enable comparability with most previous psychosis-related microstate studies (Andreou et al. 2014; Tomescu et al. 2014, 2015; da Cruz et al. 2020; de Bock et al. 2020; Murphy et al. 2020). Alternative clustering solutions investigating more classes were not conducted. A group mean for every class was computed using a permutation algorithm that minimizes common variance across subjects (Koenig et al. 1999). These group mean maps were class-labelled based on model map norms (Koenig et al. 2002) using minimal Global Map Dissimilarity. Class-labelled group mean maps were used to sort the individual microstate mean maps based on spatial similarity, quantified using topographic correlations. Microstates in individual subjects were labelled to its corresponding class based on these sorted individual means (for the rationale behind this proceeding see Supplementary Methods). Only non-truncated microstate parameters were computed, thus first and last segments were ignored. For each class the standard parameters coverage, duration and occurrence were calculated. In addition, the ratios of classes C/D of the standard parameters were analyzed, as well as TP. Two measures of TP were computed, the observed transitions of C → D and D → C per second (observed TP) and the difference of observed and expected transitions per second of C → D and D → C (delta TP). The expected transitions were calculated using a random transition model (Lehmann et al. 2005), which estimates the distribution of microstate transitions expected by chance while preserving the observed occurrence frequencies of the analyzed microstate classes within the specific dataset. This procedure was chosen to account for the possibility that individual differences in raw transition probabilities may be influenced by individual differences in the relative occurrence of microstate classes. For outlier detection, Mahalanobis distance was computed, which is a multidimensional measure of the distance of a given datapoint from the mean of all datapoints. The distance was computed with the stats package in R (R Core Team 2025) and included the crucial parameters of interest of this study (group, age, sex, explained variance, length of EEG sampling, percentage of artefacts, GFP, as well as coverage, duration and occurrence in each class). The cut-off for exclusion was defined as those five subjects who beside showing the greatest distance, simultaneously displayed heavily distorted microstate topographies on visual inspection (4 CHR individuals). These distorted topographies bore little resemblance to the canonical microstate maps and were likely attributable to excessive EEG artifacts.

### Statistical Analyses

All correlations were computed using Kendall’s rank correlation as the data was non-normally distributed and substantially ordinal by nature. Confidence intervals (CI) were computed using bootstrap resampling with 5000 iterations. To control for sex, age and antipsychotic medication (chlorpromazine equivalents), partial correlations were computed with these variables as covariates. First, we correlated the three standard microstate parameters (coverage, duration and occurrence) of microstate classes C and D with different symptom domains (SOPS positive/negative/disorganization/general, COPER, COGDIS, perceptive and non-perceptive symptoms). In a more explanatory manner, all investigated parameters (the standard parameters in combination with the ratios of C/D and TP of C → D and D → C) in all classes (A-D) were correlated with symptom domains. Second, we correlated the three standard microstate parameters of classes C and D with measures of cognitive domains (DST, TMT A&B, PPVT, RWT, SOPT, VLMT). In a more explanatory manner, all investigated parameters in all classes (A-D) were correlated with cognitive domains. Third, we correlated the three standard microstate parameters of classes C and D with the assessed functioning scales (GAF, GF-S, SOFAS). In a more explanatory manner, all investigated parameters in all classes (A-D) were correlated with functioning scales. All analyses were repeated in the unmedicated subsample to exclude potential effects of any psychotropic medication (including antipsychotics, antidepressants, stimulants and others, see Table 1). All results were corrected for multiple comparisons using the false discovery rate (FDR) method (Benjamini and Hochberg 1995) (symptom domains: *n* = 24 tests per class, symptom domains exploratory analysis: *n* = 24 tests per class for the standard parameters and *n* = 56 tests for the additional parameters; cognitive domains: *n* = 36 tests per class, cognitive domains exploratory analysis: *n* = 36 tests per class for the standard parameters and *n* = 84 tests for the additional parameters; functioning scales: *n* = 9 tests per class, functioning scales exploratory analysis: *n* = 9 tests per class for the standard parameters and *n* = 21 tests for the additional parameters). FDR correction was applied separately to each microstate class, as we considered the microstate classes to represent independent entities, whereas the microstate parameters and different scales were considered interdependent.

### Bayesian Statistics

In addition to classical frequentist statistics, Bayesian models were computed in order to estimate the likelihood of the presence or absence of effects. Bayesian linear regressions were conducted separately for each microstate parameter (including the C/D ratio and TP) within each class (A–D). In these models, microstate parameters were regressed on measures from symptom domains, neurocognitive domains, or functioning scales while age, sex, and chlorpromazine equivalents were included as covariates. Bayes factors were computed by comparing a full model (including the predictor of interest in addition to covariates) to a reduced model containing only the covariates. The regressions were computed using the lmBF function from the BayesFactor package in R (Morey et al. 2024). Across all models, a consistent default prior (Jeffreys-Zellner-Siow Cauchy prior with r = √2/4 ≈ 0.354) identically to the EEG predictor and all covariates was used. No correction for multiple comparisons was applied, as this is not recommended in Bayesian analyses (Gelman et al. 2012). Bayes factor (BF) values indicate how many times more likely the observations are under the alternative relative to the null hypothesis. According to common guidelines, BF values > 3 provide “moderate” support for the alternative hypothesis, and BF values > 10 indicate “strong” support. On the other hand, BF values < 0.33 provide “moderate,” and BF values < 0.1 provide “strong” support for the null hypothesis (van Doorn et al. 2021).

## Results

**Table 1:** Sociodemographic and clinical characteristics of the CHR sample

Sociodemographic characteristics and medication^A^			
Age in years (mean ± SD, range)		19.0 ± 4.3 (9–35)	
Age (underage/adult, n)		52/48	
Sex (f/m, n)		45/55	
Psychotropic medication (n, %)		28 (28%)	
Antidepressants (n, %)		14 (14%)	
Antipsychotics (n, %)		13 (13%)	
Stimulants (n, %)		4 (4%)	
Other psychotropic drugs (n, %)^B^		6 (6%)	
CPZ equivalents in mg (mean ± SD, range)		35.9 ± 135.5 (0-900)	
***Distribution of CHR criteria (n***,*** %)***			
BS criteria only		26 (26%)	
UHR criteria only		25 (25%)	
BS and UHR criteria		49 (49%)	
***Diagnoses and comorbidities (ICD-10)***,*** (n***,*** %)***^C^			
F10-F19	6 (6%)	F50-F59	3 (3%)
F20-F29	3 (3%)^D^	F60-F69	7 (7%)
F30-F39	50 (50%)	F80-F89	2 (2%)
F40-F49	42 (42%)	F90-F99	8 (8%)

*Abbreviations*: *BS* basic symptom, *CHR* clinical high-risk, *CPZ* chlorpromazine, *f* female, *m* male, *SD* standard deviation, *UHR* ultra-high-risk

^A^Some CHR individuals took more than one type of psychotropic medication, therefore percentages do not sum up to overall psychotropic medication

^B^Other psychotropic drugs include mood stabilizers, anticonvulsants and St. John’s wort

^C^The diagnoses are not mutually exclusive, therefore percentages overlap

^D^The three CHR individuals with F20-29 were diagnosed with schizotypal personality disorder (F21). Note that this table shows the full sample and that due to missing values in the assessments of symptoms, cognitive functions and/or social/global functioning in few CHR individuals, subsets of this full sample were analyzed. 93 CHR were assessed for correlations with symptom domains (65 unmedicated CHR), 93 CHR for cognitive functions (66 unmedicated CHR) and 98 CHR for global/social functioning (70 unmedicated CHR)

### Microstates

**Fig. 1:**
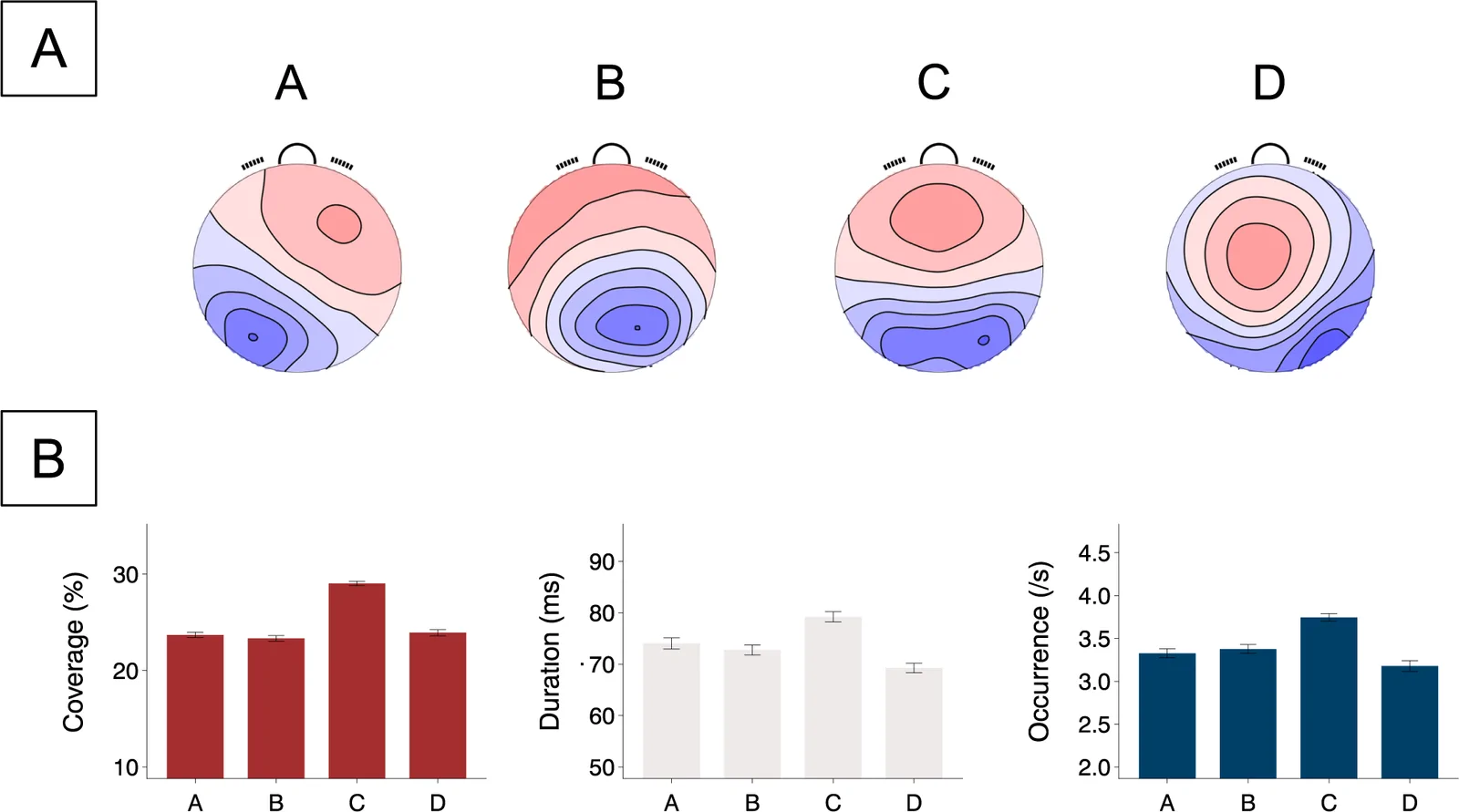
Microstate topographies and parameters. **A** Topographic maps of microstate classes A-D in the CHR group. Color steps are presented in arbitrary units, red and blue colors indicate opposite polarities. **B** Standard microstate parameters coverage, duration and occurrence in classes A-D in the CHR group

In CHR individuals microstate maps resemble the four maps consistently reported in previous literature (Michel and Koenig 2018). The topographies and microstate measures are displayed in Fig. 1. Explained variance was high (80%), with the normal range being between 65 and 84% (Michel and Koenig 2018). The mean artifact-free EEG length available for microstate analyses was 386 s (standard deviation (SD): 43 s, range: 222–496 s). In the previous analysis of the current sample (Liebrand et al. 2024), age was not significantly associated with microstate parameters, either when included as a covariate in the linear mixed-effects models or when the sample was stratified into underage and adult groups. Bayesian statistics provided moderate evidence against an age effect. Furthermore we did not detect effects of psychotropic medication status or of chlorpromazine equivalents.

### Neurocognitive Domains and Psychosocial Functioning Scales

An overview of the mean, SD, and range of the results from the instruments used to assess neurocognitive performance and psychosocial functioning is provided in Supplementary Table S4.

### Correlations of Microstates and Symptom Domains

**Fig. 2:**
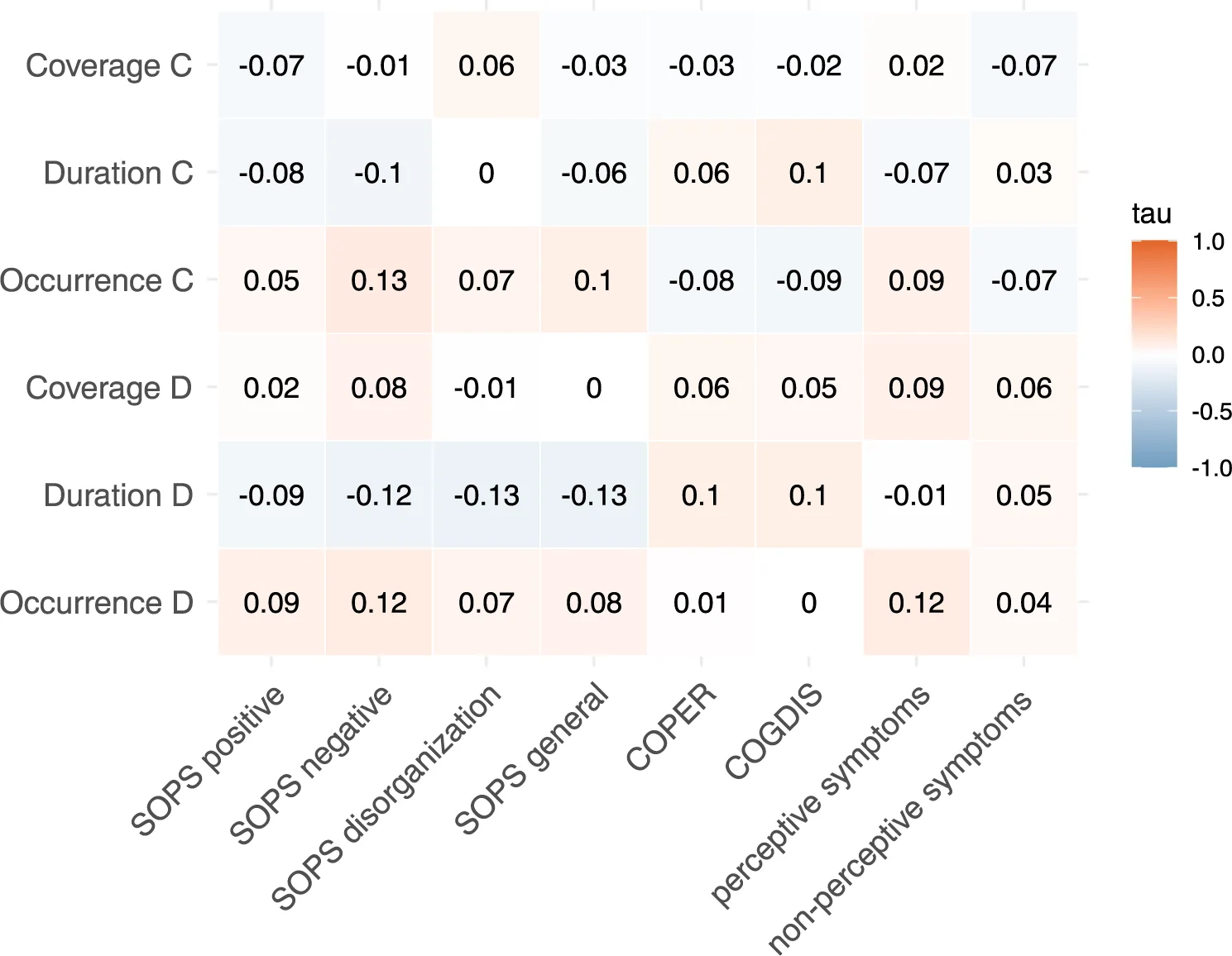
Correlation coefficients relating symptom domains to microstate parameters in classes C and D within the CHR group. There were no significant correlations. Abbreviations: COGDIS = cognitive disturbances, COPER = cognitive-perceptive basic symptoms, SOPS = Scale of Psychotic Symptoms, tau = Kendall’s rank correlation coefficient (τ)

For both classes C and D, no significant correlation was found between symptom domains and standard microstate parameters (coverage, duration, occurrence), either after FDR correction or in uncorrected results (Class C: all τ < 0.14, uncorrected *p* > 0.062, p-FDR > 0.59, BF: 0.37 to 1.24; Class D: all τ < 0.13, uncorrected *p* > 0.069, p-FDR > 0.51, BF: 0.40 to 2.22). This was also true for the subset (*n* = 65) of unmedicated CHR participants (Class C: all τ < 0.17, uncorrected *p* > 0.053, p-FDR > 0.60, BF: 0.40 to 1.06; Class D: all τ < 0.15, uncorrected *p* > 0.074, p-FDR > 0.60, BF: 0.41 to 1.98). In a more exploratory way, we correlated the standard microstate parameters in all classes (A-D), the ratios C/D, and TP (C→D, D→C) with symptom domains. When not correcting for multiple comparisons two significant correlations were found (SOPS disorganization and duration of class B: τ = -0.15, CI = -0.30 to 0.0, *p* = 0.038; SOPS general and occurrence of class A: τ = 0.15, CI = 0.0 to 0.29, *p* = 0.042), which did not survive correction for multiple comparisons (all p-FDR > 0.50). In the exploratory correlations analyzing the unmedicated CHR subgroup, two marginal significant correlations were found (SOPS disorganization and duration of class B: τ = -0.17, CI = -0.37 to 0.0, *p* = 0.043; SOPS disorganization and duration of C/D ratio: τ = 0.17, CI = 0.0 to 0.32, *p* = 0.050), which also did not survive correction for multiple comparisons (all p-FDR > 0.45). In the exploratory analyses, BF ranged between 0.37 and 3.59 for the full CHR sample and between 0.40 and 1.98 for the unmedicated subgroup. Results are displayed in Fig. 2 and Supplementary Figure S1.

### Correlations of Microstates and Neurocognitive Measures

**Fig. 3:**
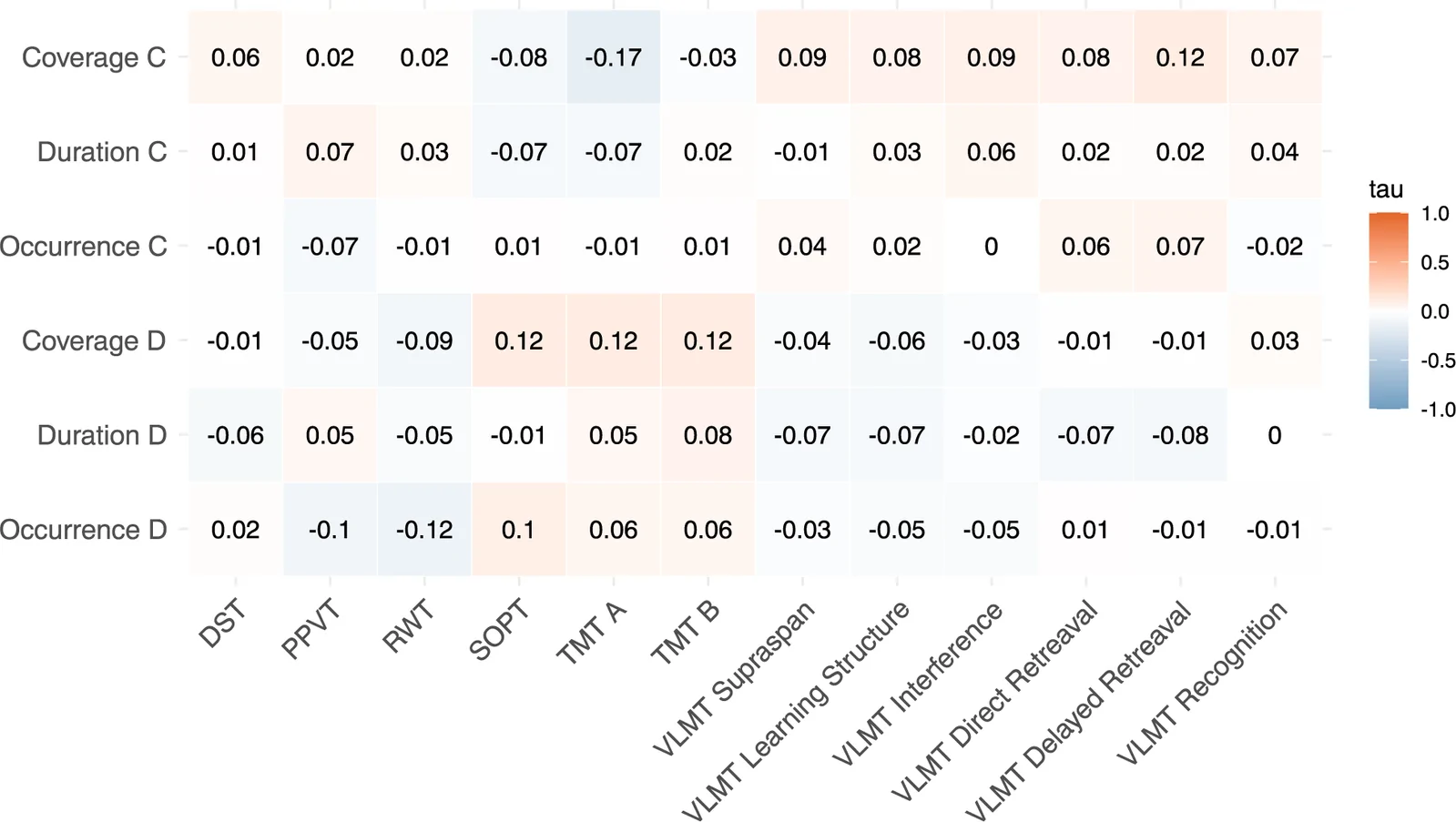
Correlation coefficients relating neurocognitive variables to microstate parameters in classes C and D within the CHR group. The correlation between TMT A and coverage of class C was significant but did not survive correction for multiple comparisons. Abbreviations: DST = Digit Symbol Task, PPVT = Peabody Picture Vocabulary Test, RWT = Regensburger Wortschatz Test, SOPT = Self-Ordered Pointing Task, tau = Kendall’s rank correlation coefficient (τ), TMT = Trail Making Task (version A and B), VLMT = Verbaler Lern- und Merkfähigkeitstest

For both classes C and D, no significant correlation was found between neurocognitive measures and standard microstate parameters (coverage, duration, occurrence) in FDR corrected analyses (Class C: all τ < 0.18, p-FDR > 0.93, BF: 0.36 to 2.35; class D: all τ < 0.13, p-FDR > 0.83, BF: 0.39 to 3.07). When not correcting for multiple comparisons, one significant correlation between neurocognitive measures and standard microstate parameters was found in class C (TMT-A and coverage in class C: τ = -0.17, CI = -0.30 to 0.0, uncorrected *p* = 0.015, p-FDR = 0.55, BF = 1.71). There was also no significant finding for the subset (*n* = 66) of unmedicated CHR participants (Class C: all τ < 0.17, uncorrected *p* > 0.053, p-FDR > 0.81, BF: 0.34 to 1.34; Class D: all τ < 0.16, uncorrected *p* > 0.072, p-FDR > 0.56, BF: 0.39 to 3.54). In a more exploratory way, we correlated the standard microstate parameters in all classes (A-D), the ratios C/D, and TP (C→D, D→C) with neurocognitive measures. When not correcting for multiple comparisons eight significant correlations were found (TMT-A & coverage class C: τ = -0.17, CI = -0.30 to 0.0, *p* = 0.015; TMT-A & coverage C/D ratio: τ = -0.17, CI = -0.31 to 0.0, *p* = 0.020; TMT-A & duration C/D ratio: τ = -0.15, CI = -0.31 to 0.0, *p* = 0.036; VLMT Interference and coverage class A: τ = 0.14, CI = 0.0 to 0.29, *p* = 0.048; VLMT Interference and coverage class B: τ = -0.15, CI = -0.28 to 0.0, *p* = 0.041; VLMT Recognition and coverage class A: τ = -0.18, CI = -0.31 to 0.0, *p* = 0.015; VLMT Recognition and coverage class B: τ = 0.14, CI = 0.0 to 0.31, *p* = 0.048; PPVT & delta TP of C→D; τ = -0.14, CI = -0.28 to 0.0, *p* = 0.045), which all did not survive correction for multiple comparisons (p-FDR > 0.51). In the exploratory correlations analyzing the unmedicated CHR subgroup eight significant correlations emerged (VLMT Learning Structure and coverage C/D ratio: τ = 0.20, CI = 0.0 to 0.35, *p* = 0.021; VLMT Learning Structure and duration C/D ratio: τ = 0.18, CI = 0.0 to 0.34, *p* = 0.037; VLMT Learning Structure and occurrence C/D ratio: τ = 0.19, CI = 0.0 to 0.34, *p* = 0.026; VLMT Recognition and coverage class B: τ = 0.25, CI = 0.0 to 0.42, *p* = 0.003; TMT-A and duration C/D ratio: τ = -0.19, CI = -0.36 to 0.0, *p* = 0.029; TMT-B and duration C/D ratio: τ = -0.17, CI = -0.33 to 0.0, *p* = 0.047; RWT and coverage class A: τ = 0.17, CI = 0.0 to 0.31, *p* = 0.049; DST and duration C/D ratio: τ = 0.20, CI = 0.0 to 0.37, *p* = 0.019), which also did not survive correction for multiple comparisons (p-FDR > 0.12). In the exploratory analyses, BF ranged between 0.34 and 8.02 for the full CHR sample and between 0.34 and 4.50 for the unmedicated subgroup. Results are displayed in Fig. 3 and Supplementary Figure S2.

### Correlations of Microstates and Psychosocial Functioning

**Fig. 4:**
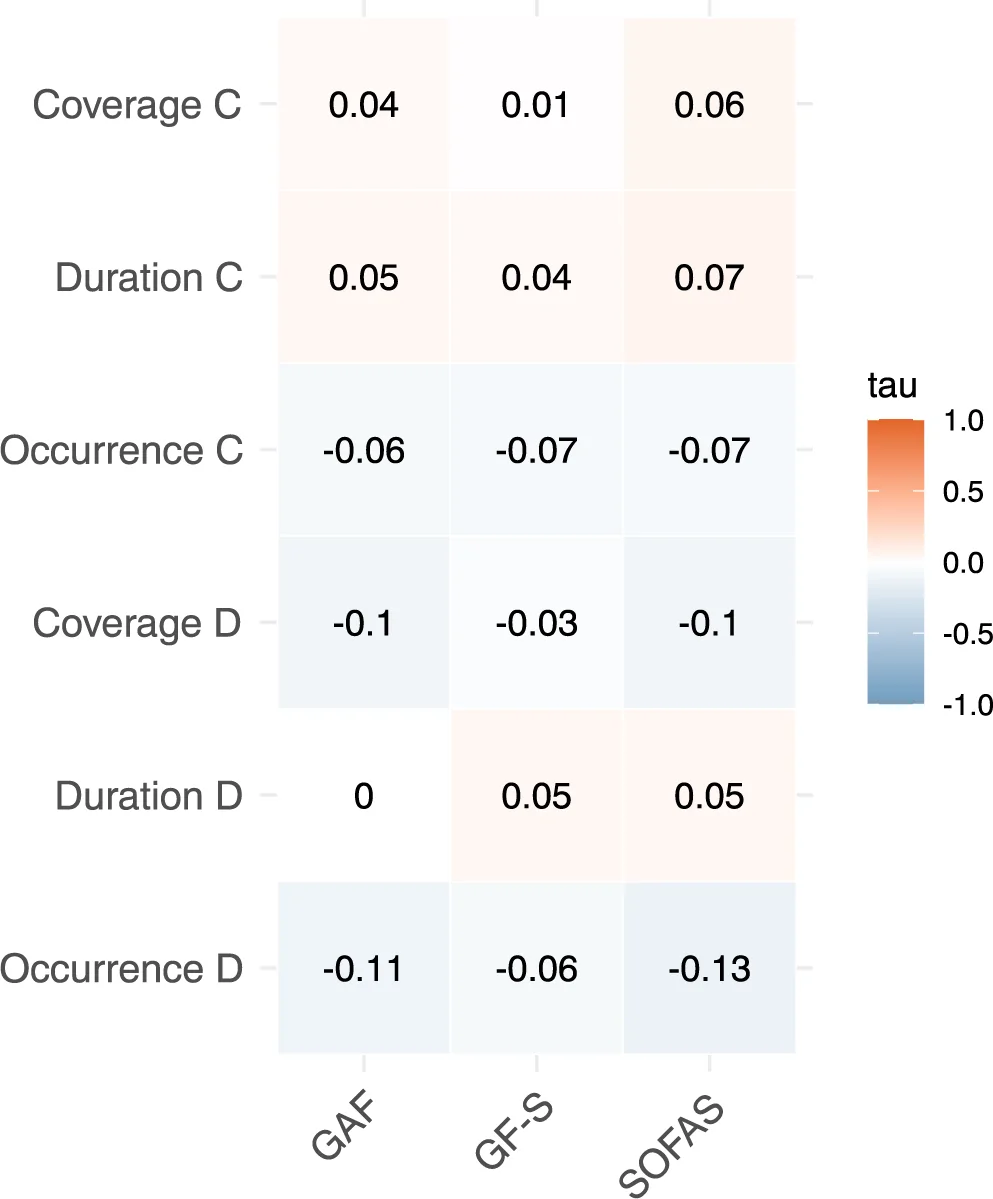
Correlation coefficients relating psychosocial functioning scales and microstate parameters in classes C and D within the CHR group. There were no significant correlations. Abbreviations: GAF = Global Assessment of Functioning, GF-S = Global Functioning: Social Scale, SOFAS = Social and Occupational Functioning Assessment Scale, tau = Kendall’s rank correlation coefficient (τ)

For both classes C and D, no significant correlation was found between psychosocial functioning scales and standard microstate parameters (coverage, duration, occurrence), either after FDR correction or in uncorrected results (class C: all τ < 0.08, uncorrected *p* > 0.290, p-FDR > 0.64, BF: 0.39 to 0.59; class D: all τ < 0.14, uncorrected *p* > 0.061, p-FDR > 0.33, BF: 0.37 to 1.35). This was also true for the subset (*n* = 70) of unmedicated CHR participants (Class C: all τ < 0.12, uncorrected *p* > 0.17, p-FDR > 0.79, BF: 0.41 to 0.54; Class D: all τ < 0.13, uncorrected *p* > 0.12, p-FDR > 0.42, BF: 0.40 to 1.86). In a more exploratory way, we correlated the standard microstate parameters in all classes (A-D), the ratios C/D, and TP (C→D, D→C) with psychosocial functioning scales. When not correcting for multiple comparisons one marginal significant correlation was found (SOFAS and occurrence ratio C/D: τ = 0.14, CI = 0.0 to 0.28, *p* = 0.041), which did not survive correction for multiple comparisons (p-FDR = 0.60). In the exploratory correlations analyzing the unmedicated CHR subgroup, three marginal significant correlations were found (GAF and observed TP of D→C: τ = -0.17, CI = -0.30 to 0.0, *p* = 0.046; GF-S and coverage of class B: τ = 0.18, CI = 0.0 to 0.33, *p* = 0.033; GF-S and duration of class B: τ = 0.17, CI = 0.0 to 0.35, *p* = 0.043), which also did not survive correction for multiple comparisons (p-FDR > 0.18). In the exploratory analyses, BF ranged between 0.37 and 2.46 for the full CHR sample and between 0.40 and 5.56 for the unmedicated subgroup. Results are displayed in Fig. 4 and Supplementary Figure S3.

## Discussion

In the present study we aimed at establishing a connection between aberrant microstate parameters and clinical symptomatology of psychosis in a large sample of 100 CHR individuals. Clinical manifestations included symptom domains, measures of neurocognitive functions and psychosocial functioning. However, despite the large sample size, entailing a substantial subgroup of unmedicated individuals at CHR, no robust associations could be established.

Interestingly, in a recent study investigating a large group of healthy subjects, similarly no connections between microstates C and D and a wide battery of cognitive functions were found (Chenot et al. 2024). However, when narrowing it down to the CHR state, our null findings contrast with those of the only two studies to date that have examined associations between clinical measures and microstate parameters in CHR individuals. One study found positive correlations between microstate A and PANSS/SOPS positive symptoms, and a negative correlation between SOPS positive symptoms and class B (Andreou et al. 2014). Note that these results should be interpreted with caution, as the sample sizes of the included groups in that study were very small. The most comprehensive microstate study of the CHR state to date, including 460 CHR individuals, found that temporal stability of microstate networks across classes was negatively associated with SOPS positive symptoms and with attentional functions (Liebrand et al. 2026). Taken together with our null finding, these mixed results do not provide evidence for consistent relationships. To resolve these heterogeneous outcomes, further large-scale studies in CHR populations are needed to investigate associations between microstate characteristics and clinical symptomatology.

One of the promises of cognitive neuroimaging is that it leads to stable, clinically applicable and reproducible biomarkers classifying and possibly predicting the course of mental disorders, including the CHR state and schizophrenia (Aydin et al. 2019; Kraguljac et al. 2021; Mamah 2023). Microstates have been proposed as such a biomarker for the CHR state (de Bock et al. 2020; Liebrand et al. 2026) and schizophrenia (Yao et al. 2024). An important criterion of a valid trait biomarker is that it shows temporal stability, and thus does not fluctuate over time as symptomatology often does (Moriarity and Alloy 2021). An optimistic explanation of the current finding is therefore that microstates might classify as trait biomarker, independent of symptoms, psychosocial or cognitive functioning, all prone to state-dependent changes over time. In line with this idea, microstates generally have been interpreted as reflections of very fundamental cognitive processes, namely as “atoms of thought” (Koukkou and Lehmann 1983) or basic buildings blocks of information processing (Schiller et al. 2024), thereby potentially not being sensitive enough to capture high-order symptomatology, which is influenced by multiple internal and external variables. Further supporting this notion, a recent review summarizing the existing knowledge of the possible underlying cognitive processes (Tarailis et al. 2023), provides rather fuzzy connections between microstates and distinct cognitive processes. This might point to an absence of such direct associations. It may therefore be necessary to first gain a better understanding of the core underlying principles of microstate networks before meaningful links between microstates and cognitive processing in specific disorders or risk-states such as the CHR syndrome can be established.

Alternatively, the heterogeneity of the CHR state itself might be the cause for our null finding. The CHR state has been proposed as a transdiagnostic risk stage, leading to multiple possible endpoints including schizophrenia and bipolar disorder, but also depression, personality disorders and substance misuse (McGorry et al. 2018). CHR individuals therefore may represent a very diverse group, with some of them progressing to one of several psychiatric disorders, while others will not develop a psychiatric condition at all. Thus, symptoms and the underlying causal effects might vary to such a strong degree that cannot be captured by microstates as the neurobiological variable of interest. Aligning with this idea, a recent study did not find differences in microstate measures between several psychiatric disorders including the CHR state, FEP, schizophrenia, major depressive disorder, bipolar disorder and autism (Iftimovici et al. 2023). However, this argument is challenged by our previous finding that microstates alterations in class D coverage and occurrence might be specific to the CHR state (Liebrand et al. 2024) and two others suggesting that specific microstate characteristics are associated with future progression from the CHR state to psychosis (de Bock et al. 2020; Liebrand et al. 2026).

Bayesian analyses yielded only anecdotal or inconclusive evidence against associations between microstate parameters and clinical symptoms, neurocognitive measures, and psychosocial functioning, rather than providing moderate or strong evidence in support of the null hypothesis. Confidence intervals of the significant effects (all uncorrected) further suggest uncertainty regarding the magnitude of these associations, with lower bounds approaching zero, indicating that any effects may be small or that their precise size remains difficult to determine. Therefore, other possible explanations for the absence of a finding in this study have to be considered. First, low reliability of the microstate parameters measured in the present dataset might underlie the null finding. This could be due to generally low reliability of microstate parameters, specific decisions in preprocessing, in feature extraction or because of poor data quality. While microstate parameters have generally been shown to exhibit good reliability (Khanna et al. 2014; Popov et al. 2023; Kleinert et al. 2023), reliability may vary across datasets and can be adversely affected by factors such as small sample sizes, residual artifacts, or suboptimal microstate extraction procedures. However, several aspects speak for good quality of the present data, including the large sample size, high explained variance, close resemblance of the topographies to previous studies and a low percentage of the artifactual EEG which had to be removed. But note that the decision to use individual maps as templates for feature extraction might have introduced additional variability, thereby potentially limiting reliability. Second, clinical symptom ratings were based on personal reports and are therefore sensitive to measurement error and fluctuations over time, giving rise to reliability concerns. This drawback might be particularly relevant in a sample with a young age range as the present one. Third, the EEG assessment represents a brief snapshot of brain dynamics and may not adequately capture the temporal variability of clinical symptoms that naturally fluctuate across days or weeks. Fourth, despite its comparably large sample size, this study might be underpowered. Although the study was likely sufficiently powered to detect medium to large effect sizes, smaller yet clinically meaningful associations may have gone undetected. In line with this, brain-behavior associations have in general shown poor replicability across studies, with recent work suggesting that thousands of participants are required for replicable findings in cross-sectional designs (Spisak et al. 2023; Marek and Laumann 2025). Finally, existing brain–behavior relationships may have gone undetected because they might be nonlinear in nature and therefore cannot be captured by the correlational analyses performed here.

### Strengths and Limitations

This study incorporates some strengths highlighted by a methodological article on correlations between neurobiological markers and symptoms of schizophrenia (Mathalon and Ford 2012). These include a large enough sample size to detect medium to large correlations, a substantial medication-naïve subgroup, multi-item ratings for symptom domains and robust neurocognitive testing. The absence of findings in the unmedicated subgroup mirrored that in the full sample. However, the current study and its design also contain weaknesses to be addressed. No healthy control group was included, and the number of microstate classes was fixed at four to ensure comparability with previous studies, although more recent work suggests that a greater number of classes may provide a more nuanced characterization of the data (Michel and Bréchet 2026). Given the substantial number of tests performed, rigorous control for multiple testing was applied using FDR correction. However, this approach carries the risk that small associations were obscured. The study was conducted at a single site, and the findings were not externally validated, limiting their generalizability and reproducibility. There may also be selection bias, as CHR participants were recruited from a specialized early detection center (FETZ), potentially overrepresenting help-seeking individuals compared with the general CHR population. Finally, the cross-sectional nature of our study limits conclusions regarding the potential role of microstates as trait biomarker.

### Implications for Future Clinical Studies

To establish whether microstates indeed can be considered stable trait-related biomarkers, large-scale longitudinal studies in CHR individuals are needed (Michel and Bréchet 2026). These should demonstrate their stability over time and investigate the predictive value of microstate parameters for conversion and remission. Clinical symptomatology and psychosocial functioning may also benefit from more ecologically valid and longitudinal assessment approaches, such as ecological momentary assessments (EMA) and movement-based profiling. It will also be crucial to determine whether specific microstate alterations observed in CHR groups are specific to the CHR syndrome, rather than reflecting more broad factors such as general psychopathology or psychosocial stress. EEG microstates also have the potential to be integrated in multimodal prediction models (Koutsouleris et al. 2021; Reinen et al. 2024), which combine predictors from different modalities to optimize individualized risk stratification. Furthermore, moving beyond conventional microstate parameters, higher-order microstate characteristics, such as microstate complexity and microstate sequence analyses (Artoni et al. 2023; von Wegner et al. 2024; Haydock et al. 2025), may offer additional insights into the neural mechanisms underlying disorder-related dysfunction and potential compensatory processes.

### Conclusions

This is the first study to systematically investigate associations between microstate parameters and clinical correlates, including symptom domains, psychosocial functioning, and neurocognitive measures, in a large cohort of 100 individuals at high risk for psychosis. No significant correlations between microstate parameters and markers of clinical symptomatology could be revealed. One explanation might be that this null finding is related to the possible basic neurobiological nature of microstates, reflecting very fundamental and unspecific neural processes which have yet to be elucidated in their specific meaning.

## Supplementary Information

Supplementary Material 1.

## Data Availability

The data can be made available on reasonable request from the corresponding author.

## Abbreviations

CHR: Clinical high-risk
FEP: First episode of psychosis
CC: Clinical controls
HC: Healthy controls
BS: Basis symptoms
