# Supplementary material for: EEG Microstates are Not Associated with Psychotic Symptoms, Neurocognitive Domains or Psychosocial Functioning in Adolescents and Young Adults with Clinical High Risk for Psychosis
Source: Brain Topogr. 2026 Oct 10;39(6):101. doi: 10.1007/s10548-026-01255-x (PMC13653868; doi:10.1007/s10548-026-01255-x)
Supplement: Supplementary file 1 [file 10548_2026_1255_MOESM1_ESM.docx]

**SUPPLEMENTARY MATERIAL**

**EEG microstates are not associated with psychotic symptoms, cognitive domains or psychosocial functioning in adolescents and young adults with clinical high risk for psychosis**

M. Liebrand *et al.*

**Supplementary Methods**

**Assessment of clinical high-risk (CHR) criteria**

CHR symptoms and criteria were assessed using semi-structured interviews with good interrater reliability (Miller et al. 2002; Schultze-Lutter et al. 2007b). These interviews comprised the following:

(1) For ultra-high-risk (UHR) symptoms and criteria the Structured Interview for Psychosis-Risk Syndromes (SIPS) (McGlashan et al. 2010) was used, measuring the attenuated psychotic symptoms (APS) criterion, and the brief intermittent psychotic symptoms (BIPS) criterion. Patients exclusively displaying the requirements of the genetic risk and functional decline (GFDR) criterion were not classified as UHR.
(2) Schizophrenia Proneness Instrument, both the Adult version (SPI-A) (Schultze-Lutter et al. 2007a) and the version for Children and Adolescents (SPI-CY) (Schultze-Lutter and Koch 2010; Fux et al. 2013) were used for predictive basic symptoms (BS) and related criteria, i.e. cognitive-perceptive basic symptoms (COPER) and/or cognitive disturbances (COGDIS).

**Recoding of basic symptoms**

For the SPI-A/SPI-CY the ratings of 7 (has always been present in same severity; trait), and 9 (the presence of this basic symptom can neither be unambiguously ruled in nor out) were recoded to 0 (7 was present overall 34 times in 18 different subjects; 9 was present overall 3 times in 2 different subjects), as in both of these cases the most conservative estimate is that no psychotic symptom was present. The rating of 8 (basic symptom is definitely present, but its frequency of occurrence is unknown) has been recoded to 1 (8 was present once in 1 subject) in order to provide the most conservative estimate of this rating.

**Microstate feature extraction**

Note that in the previous study (Liebrand et al. 2024), from which the current data stems from, the mean microstate maps for all CHR individuals were sorted using an overarching mean for all groups (CHR, FEP, CC, HC). This was not applicable for the present analysis which only included the CHR sample. However, sorting the mean CHR maps using the norm maps from Koenig et al. 2002 led to the same results, which would be the valid procedure if only one group was analyzed. Therefore, microstate results from the previous study were directly used here. For labelling of microstates on the individual level, we used templates of single subjects (and not group means), acknowledging the present broad age-range, since microstates show an evolution with age (Koenig et al. 2002; Tomescu et al. 2018).

**Table S1.**	Description of the SPI-A Items

| BS 1 | Inability to divide attention (B1) |
| --- | --- |
| BS 2 | Disturbance of expressive language (C5) |
| BS 3 | Disturbance of symbol comprehension – concretism (O3) |
| BS 4 | Captivation by perceptual details (O7) |
| BS 5 | Interference of emotionally neutral thoughts (C2) |
| BS 6 | Blocking of the respective train of thought (C3) |
| BS 7 | Disturbance of receptive language (C4) |
| BS 8 | Racing and crowding of thoughts (D3) |
| BS 9 | Tendency toward self-reference (D4) |
| BS 10 | Perseveration of specific emotionally neutral mental contents (O1) |
| BS 11 | Impaired discrimination between ideas and perceptions, between imagination and memory (O2) |
| BS 12 | Derealization (O8) |
| BS 13 | Visual perception disturbances (O4, F1, F2) |
| BS 14 | Auditory perception disturbances (O5, F5) |

*Description of the used items of the Schizophrenia Proneness Instrument – Adult version (SPI-A). The respective item numbers are showed in parentheses.*

**Table S2.**	Description of the SPI-CY Items

| BS 1 | Inability to divide attention (D8) |
| --- | --- |
| BS 2 | Captivation by perceptual details (O2) |
| BS 3 | Disturbance of expressive language (D12) |
| BS 4 | Disturbance of symbol comprehension – concretism (D7) |
| BS 5 | Interference of emotionally neutral thoughts (D9) |
| BS 6 | Blocking of the respective train of thought (D15) |
| BS 7 | Racing and crowding of thoughts (D10) |
| BS 8 | Disturbance of receptive language (D11) |
| BS 9 | Tendency toward self-reference (B2) |
| BS 10 | Impaired discrimination between ideas and perceptions, between imagination and memory (B1) |
| BS 11 | Perseveration of specific emotionally neutral mental contents (D14) |
| BS 12 | Derealization (B7) |
| BS 13 | Visual perception disturbances (B3, O1, O3) |
| BS 14 | Auditory perception disturbances (B4, B5) |

*Description of the used items of the Schizophrenia Proneness Instrument –Child and Youth version (SPI-CY). The respective item numbers are showed in parentheses.*

**Table S3.**	Description of the SOPS Items

| P1 | Unusual Thought Content / Delusional Ideas |
| --- | --- |
| P2 | Suspiciousness / Persecutory Ideas |
| P3 | Grandiose Ideas |
| P4 | Perceptual Abnormalities / Hallucinations |
| P5 | Disorganized Communication |
| N1 | Social anhedonia |
| N2 | Avolition / amotivation |
| N3 | Expression of emotion (reduced emotional expression) |
| N4 | Experience of emotions and self (internal experience of emotion) |
| N5 | Ideational richness / impoverished thinking |
| N6 | Occupational functioning |
| D1 | Odd behavior or appearance |
| D2 | Bizarre thinking |
| D3 | Trouble with focus and attention |
| D4 | Personal hygiene / social functioning decline |
| G1 | Sleep disturbance |
| G2 | Dysphoric mood |
| G3 | Motor disturbances |
| G4 | Impaired tolerance to normal stress |

*Description of the used items of the Scale of Prodromal Symptoms (SOPS), including the positive (P), negative (N), disorganization (D) and general (G) subscales.*

**Supplementary Results**

**Table S4.**	Descriptive statistics of the results from the instruments used to assess neurocognition and psychosocial functioning

| ***Clinical measure*** | ***Mean, SD, range*** |
| --- | --- |
| **DST** (raw values) | 9.6±2.8 (2-19) |
| **PPVT** (percentile ranks) | 60.8±27.6 (2-100) |
| **RWT** (a mean of the percentile ranks over fruits, sports, animals, s-words) | 35.1±19.1 (2.7-83.3) |
| **SOPT** (number of errors) | 4.7±3.1 (0-13) |
| **TMT A** (time in s) | 26.8±10.2 (10-71) |
| **TMT B** (time in s) | 64.3±25.4 (16-149) |
| **VLMT Supraspan** (percentile ranks) | 47.3±30.3 (5-95) |
| **VLMT Learning Structure** (percentile ranks) | 56.4±30.4 (5-95) |
| **VLMT Intereference** (percentile ranks) | 40.8±32.0 (5-95) |
| **VLMT Direct Retrieval** (percentile ranks) | 51.0±30.0 (5-95) |
| **VLMT Delayed Retrieval** (percentile ranks) | 54.6±30.7 (5-95) |
| **VLMT Recognition** (raw values) | 14.5±0.9 (11-15) |
| **GAF** (raw values) | 53.0±11.7 (21-78) |
| **GF-S** (raw values) | 6.8±1.4 (3-9) |
| **SOFAS** (raw values) | 61.0±12.6 (35-85) |

*Mean, standard deviation (SD), and range of the instruments used to assess neurocognition and psychosocial functioning, which were correlated with microstate measures. Abbreviations: DST = Digit Symbol Task; PPVT = Peabody Picture Vocabulary Test; RWT = Regensburger Wortschatz Test; SOPT = Self-Ordered Pointing Task; TMT = Trail Making Task (version A and B); VLMT = Verbaler Lern- und Merkfähigkeitstest; GAF = Global Assessment of Functioning; GF-S = Global Functioning: Social Scale; SOFAS = Social and Occupational Functioning Assessment Scale.*

**Fig. S1**	***Correlation coefficients from the exploratory analysis relating symptom domains to all investigated microstate metrics in all classes within the CHR group.*** *No correlation was significant after correction for multiple comparisons. The two correlations being significant in the uncorrected results are highlighted with a black square. Abbreviations: COGDIS = cognitive disturbances, COPER = cognitive-perceptive basic symptoms, SOPS = Scale of Psychotic Symptoms, tau = Kendall’s rank correlation coefficient (τ), TP = transition probabilities.*

**Fig. S2	*Correlation coefficients relating neurocognitive variables to all investigated microstate metrics in all classes within the CHR group****. No correlation was significant after correction for multiple comparisons. The eight correlations being significant in the uncorrected results are highlighted with a black square. Abbreviations: DST = Digit Symbol Task, PPVT = Peabody Picture Vocabulary Test, RWT = Regensburger Wortschatz Test, SOPT = Self-Ordered Pointing Task, tau = Kendall’s rank correlation coefficient (τ), TMT = Trail Making Task (version A and B), TP = transition probabilities, VLMT = Verbaler Lern- und Merkfähigkeitstest.*

**Fig. S3	*Correlation coefficients relating psychosocial functioning scales and all investigated microstate metrics in all classes within the CHR group.*** *No correlation was significant after correction for multiple comparisons. The single correlation being significant in the uncorrected results is highlighted with a black square. Abbreviations: GAF = Global Assessment of Functioning, GF-S = Global Functioning: Social Scale, SOFAS = Social and Occupational Functioning Assessment Scale, tau = Kendall’s rank correlation coefficient (τ), TP = transition probabilities.*

**Supplementary References**

Fux L, Walger P, Schimmelmann BG, Schultze-Lutter F (2013) The Schizophrenia Proneness Instrument, Child and Youth version (SPI-CY): Practicability and discriminative validity. Schizophrenia Research 146:69–78. https://doi.org/10.1016/j.schres.2013.02.014

Koenig T, Prichep L, Lehmann D, et al (2002) Millisecond by Millisecond, Year by Year: Normative EEG Microstates and Developmental Stages. NeuroImage 16:41–48. https://doi.org/https://doi.org/10.1006/nimg.2002.1070

Liebrand M, Katsarakis A, Josi J, et al (2024) EEG microstate D as psychosis-specific correlate in adolescents and young adults with clinical high risk for psychosis and first-episode psychosis. Schizophrenia Research 264:49–57. https://doi.org/10.1016/j.schres.2023.11.014

McGlashan T, Cannon T, Walsh B, Woods S (2010) The Psychosis-Risk Syndrome: Handbook for Diagnosis and Follow-Up, 1st edn. Oxford University Press, Oxford

Miller TJ, McGlashan TH, Rosen JL, et al (2002) Prospective Diagnosis of the Initial Prodrome for Schizophrenia Based on the Structured Interview for Prodromal Syndromes: Preliminary Evidence of Interrater Reliability and Predictive Validity. AJP 159:863–865. https://doi.org/10.1176/appi.ajp.159.5.863

Schultze-Lutter F, Addington J, Rurhmann S (2007a) Schizophrenia Proneness Instrument, Adult Version (SPI-A), 1st edn. Giovanni Fioriti Editore, Rome

Schultze-Lutter F, Klosterkötter J, Picker H, et al (2007b) Predicting first-episode psychosis by basic symptom criteria. Clinical Neuropsychiatry: Journal of Treatment Evaluation 4:11–22

Schultze-Lutter F, Koch E (2010) Schizophrenia Proneness Instrument: child and youth version (SPI-CY). Fioriti Rome

Tomescu MI, Rihs TA, Rochas V, et al (2018) From swing to cane: Sex differences of EEG resting-state temporal patterns during maturation and aging. Developmental Cognitive Neuroscience 31:58–66. https://doi.org/https://doi.org/10.1016/j.dcn.2018.04.011
